# 2-(5-Bromo­pyridin-3-yl)-5-[3-(4,5,6,7-tetra­hydro­thieno[3,2-*c*]pyridine-5-ylsulfon­yl)thio­phen-2-yl]-1,3,4-oxa­diazole

**DOI:** 10.1107/S1600536811038529

**Published:** 2011-09-30

**Authors:** Hoong-Kun Fun, Madhukar Hemamalini, Sankappa Rai, A. M. Isloor, Prakash Shetty

**Affiliations:** a X-ray Crystallography Unit, School of Physics, Universiti Sains Malaysia, 11800 USM, Penang, Malaysia; b Department of Chemistry, Manipal Institute of Technology, Manipal, India; c Medicinal Chemistry Division, Department of Chemistry, National Institute of Technology, Karnataka, Surathkal, Mangalore 575 025, India; d Department of Printing & Media, Manipal Institute of Technology, Manipal, India

## Abstract

In the title compound, C_18_H_13_BrN_4_O_3_S_3_, the tetra­hydro­pyridine ring adopts a half-chair conformation with the central methyl­ene-C atom of the NCH_2_CH_2_ unit at the flap. The dihedral angles between the tetra­hydro­pyridine ring and the pyridine and two thio­phene rings are 69.34 (13) 5.66 (13) and 68.63 (13)°, respectively, while the dihedral angle between the 1,3,4-oxadiazole and tetra­hydro­pyridine rings is 54.76 (13)°. The mol­ecule is stabilized by an intra­molecular C—H⋯N inter­action. In the crystal, adjacent mol­ecules are connected *via* bifurcated C—H⋯(N,O) hydrogen bonds, forming a chain along the *b* axis.

## Related literature

For applications of 4,5,6,7-tetra­hydro­thieno[3,2-*c*]pyridine derivatives, see: Lopez-Rodriguez *et al.* (2001 ▶); Roth *et al.* (1994 ▶); Ying & Rusak (1997 ▶). For ring conformational analysis, see: Cremer & Pople (1975 ▶).
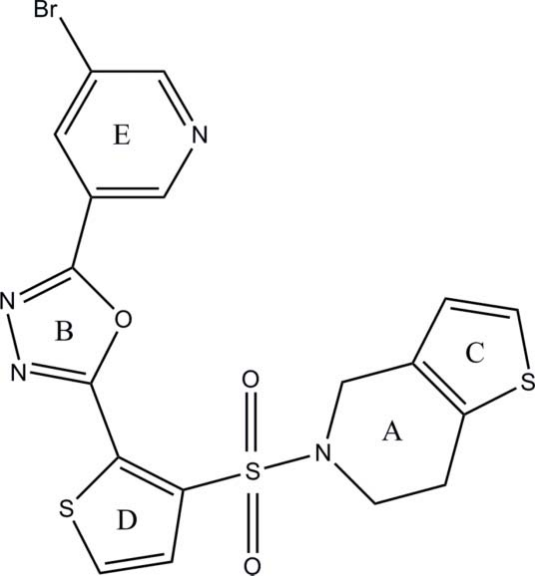


## Experimental

### 

#### Crystal data

C_18_H_13_BrN_4_O_3_S_3_
*M* *_r_* = 509.41
Monoclinic, 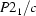

*a* = 7.0327 (14) Å
*b* = 7.6488 (15) Å
*c* = 36.939 (7) Å
β = 91.315 (5)°
*V* = 1986.5 (7) Å^3^
*Z* = 4
Mo *K*α radiation
μ = 2.41 mm^−1^
*T* = 296 K
0.35 × 0.13 × 0.05 mm

#### Data collection

Bruker APEXII DUO CCD area-detector diffractometer
Absorption correction: multi-scan (*SADABS*; Bruker, 2009 ▶) *T*_min_ = 0.482, *T*_max_ = 0.885
22958 measured reflections
7230 independent reflections
4160 reflections with *I* > 2σ(*I*)
*R*_int_ = 0.051

#### Refinement

*R*[*F* ^2^ > 2σ(*F* ^2^)] = 0.046
*wR*(*F* ^2^) = 0.123
*S* = 1.02
7230 reflections
262 parameters
H-atom parameters constrained
Δρ_max_ = 0.76 e Å^−3^
Δρ_min_ = −0.73 e Å^−3^

### 

Data collection: *APEX2* (Bruker, 2009 ▶); cell refinement: *SAINT* (Bruker, 2009 ▶); data reduction: *SAINT*; program(s) used to solve structure: *SHELXTL* (Sheldrick, 2008 ▶); program(s) used to refine structure: *SHELXTL*; molecular graphics: *SHELXTL*; software used to prepare material for publication: *SHELXTL* and *PLATON* (Spek, 2009 ▶).

## Supplementary Material

Crystal structure: contains datablock(s) global, I. DOI: 10.1107/S1600536811038529/tk2791sup1.cif

Structure factors: contains datablock(s) I. DOI: 10.1107/S1600536811038529/tk2791Isup2.hkl

Supplementary material file. DOI: 10.1107/S1600536811038529/tk2791Isup3.cml

Additional supplementary materials:  crystallographic information; 3D view; checkCIF report

## Figures and Tables

**Table 1:** Hydrogen-bond geometry (Å, °)

*D*—H⋯*A*	*D*—H	H⋯*A*	*D*⋯*A*	*D*—H⋯*A*
C7—H7*A*⋯N2	0.97	2.52	3.283 (4)	136
C10—H10*A*⋯O3^i^	0.93	2.49	3.330 (3)	150
C10—H10*A*⋯N2^i^	0.93	2.42	3.183 (3)	139

Symmetry code: (i) 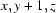.

**Fig. 1.**
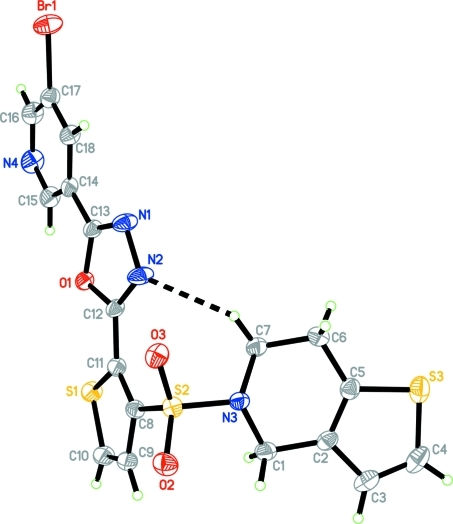
The molecule of the title compound, showing 30% probability displacement ellipsoids. The dashed line represents a C—H···N interaction.

**Fig. 2.**
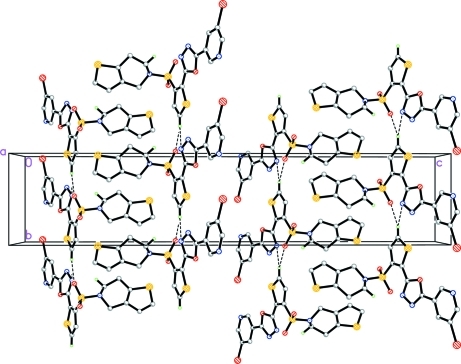
A view of the crystal packing for the title compound (I). The dashed lines represent C—H···O and C—H···N hydrogen bonds.

## supplementary crystallographic information

### Comment

4,5,6,7-Tetrahydrothieno[3,2-c]pyridine derivatives are extensively studied in medicinal chemistry due to their various biological activities (Lopez-Rodriguez *et al.*, 2001). 4,5,6,7-Tetrahydrothieno[3,2-c] pyridine oxadiazole derivatives are mainly used in CNS functions and disorders such as schizophrenia (Roth *et al.*, 1994), depression, epilepsy, migraine, and control of circadian rhythm (Ying & Rusak, 1997).

The molecular structure of the title compound, Fig. 1, contains five rings, namely, A (N3/C1,C2,C5–C7), B (N1/N2/O1/C12,C13), C (S3/C2–C5), D (S1/C8–C11) and E (N4/C14–C18). The tetrahydropyridine (N3/C1,C2,C5–C7) ring adopts a half-chair conformation with puckering parameters Q = 0.497 (3) Å, θ = 131.5 (3)° and φ = 141.6 (4)°  with the flap atom at C7 [maximum deviation of -0.338 (3) Å]. The dihedral angle between the least-squares planes of the rings are A/B = 54.76 (13)°, A/C = 5.66 (13)°, A/D = 68.63 (13)°, A/E = 69.34 (13)°, B/C = 56.97 (14)°, B/D = 13.90 (14)°, B/E = 15.62 (13)°, C/D = 70.85 (13)°, and C/E = 70.88 (13)°.

In the crystal structure, (Fig. 2), adjacent molecules are connected *via* bifurcated C—H···N and C—H···O (Table 1) hydrogen bonds forming one-dimensional chains along the *b*-axis.

### Experimental

To a mixture of 3-(6,7-dihydrothieno[3,2-c]pyridine-5(4*H*)-ylsulfonyl) thiophene-2-carbohydrazide (0.5 g, 0.0014 mol) and 5-bromopyridine-3-carboxylic acid (0.29 g, 0.0014 mol), neutral alumina (0.5 g) and POCl_3_, (1.1 g, 0.007 mol) were added. The resulting mixture was irradiated in a microwave oven for 5 min. Mass analysis of the crude reaction mixture confirmed completion of the reaction. The reaction mixture was concentrated and the residue was purified by column chromatography to get title compound which was recrystallised using acetone. Yield: 68%, m.p. 429–431 K.

### Refinement

All hydrogen atoms were positioned geometrically [C–H = 0.93 or 0.97 Å] and were refined using a riding model, with *U*_iso_(H) = 1.2 *U*_eq_(C).

### Crystal data

**Table:**

C_18_H_13_BrN_4_O_3_S_3_	*F*(000) = 1024
*M**_r_* = 509.41	*D*_x_ = 1.703 Mg m^−^^3^
Monoclinic, *P*2_1_/*c*	Mo *K*α radiation, λ = 0.71073 Å
Hall symbol: -P 2ybc	Cell parameters from 3605 reflections
*a* = 7.0327 (14) Å	θ = 2.7–25.9°
*b* = 7.6488 (15) Å	µ = 2.41 mm^−^^1^
*c* = 36.939 (7) Å	*T* = 296 K
β = 91.315 (5)°	Plate, colourless
*V* = 1986.5 (7) Å^3^	0.35 × 0.13 × 0.05 mm
*Z* = 4	

### Data collection

**Table:**

Bruker APEXII DUO CCD area-detector diffractometer	7230 independent reflections
Radiation source: fine-focus sealed tube	4160 reflections with *I* > 2σ(*I*)
graphite	*R*_int_ = 0.051
φ and ω scans	θ_max_ = 32.7°, θ_min_ = 2.2°
Absorption correction: multi-scan (*SADABS*; Bruker, 2009)	*h* = −10→8
*T*_min_ = 0.482, *T*_max_ = 0.885	*k* = −11→11
22958 measured reflections	*l* = −55→52

### Refinement

**Table:**

Refinement on *F*^2^	Primary atom site location: structure-invariant direct methods
Least-squares matrix: full	Secondary atom site location: difference Fourier map
*R*[*F*^2^ > 2σ(*F*^2^)] = 0.046	Hydrogen site location: inferred from neighbouring sites
*wR*(*F*^2^) = 0.123	H-atom parameters constrained
*S* = 1.02	*w* = 1/[σ^2^(*F*_o_^2^) + (0.0445*P*)^2^ + 0.8759*P*] where *P* = (*F*_o_^2^ + 2*F*_c_^2^)/3
7230 reflections	(Δ/σ)_max_ < 0.001
262 parameters	Δρ_max_ = 0.76 e Å^−^^3^
0 restraints	Δρ_min_ = −0.73 e Å^−^^3^

### Special details

**Table:**

Geometry. All s.u.'s (except the s.u. in the dihedral angle between two l.s. planes) are estimated using the full covariance matrix. The cell s.u.'s are taken into account individually in the estimation of s.u.'s in distances, angles and torsion angles; correlations between s.u.'s in cell parameters are only used when they are defined by crystal symmetry. An approximate (isotropic) treatment of cell s.u.'s is used for estimating s.u.'s involving l.s. planes.
Refinement. Refinement of F^2^ against ALL reflections. The weighted R-factor wR and goodness of fit S are based on F^2^, conventional R-factors R are based on F, with F set to zero for negative F^2^. The threshold expression of F^2^ > 2σ(F^2^) is used only for calculating R-factors(gt) etc. and is not relevant to the choice of reflections for refinement. R-factors based on F^2^ are statistically about twice as large as those based on F, and R- factors based on ALL data will be even larger.

### Fractional atomic coordinates and isotropic or equivalent isotropic displacement parameters (Å^2^)

**Table:**

	*x*	*y*	*z*	*U*_iso_*/*U*_eq_	
S1	0.93996 (10)	0.96690 (8)	0.10025 (2)	0.04559 (16)	
S2	1.36853 (8)	0.62674 (8)	0.146401 (17)	0.03714 (14)	
S3	1.17503 (12)	0.51687 (12)	0.30392 (2)	0.0593 (2)	
Br1	0.24445 (4)	−0.00049 (4)	0.017218 (10)	0.06497 (12)	
O1	0.7735 (2)	0.6343 (2)	0.07860 (4)	0.0371 (4)	
O2	1.5532 (2)	0.7035 (3)	0.14769 (5)	0.0505 (5)	
O3	1.3410 (3)	0.4632 (2)	0.12866 (5)	0.0464 (4)	
N1	0.7985 (3)	0.3595 (3)	0.09508 (7)	0.0503 (6)	
N2	0.9456 (3)	0.4530 (3)	0.11199 (7)	0.0513 (6)	
N3	1.3043 (3)	0.6029 (3)	0.18809 (5)	0.0358 (4)	
N4	0.2436 (3)	0.5346 (4)	0.02560 (8)	0.0582 (6)	
C1	1.3498 (4)	0.7466 (3)	0.21341 (7)	0.0443 (6)	
H1A	1.2736	0.8485	0.2073	0.053*	
H1B	1.4829	0.7781	0.2117	0.053*	
C2	1.3091 (3)	0.6885 (3)	0.25127 (7)	0.0397 (5)	
C3	1.3612 (4)	0.7808 (4)	0.28326 (8)	0.0555 (7)	
H3A	1.4313	0.8839	0.2833	0.067*	
C4	1.2989 (4)	0.7039 (5)	0.31349 (9)	0.0619 (8)	
H4A	1.3206	0.7472	0.3368	0.074*	
C5	1.2078 (3)	0.5430 (4)	0.25829 (7)	0.0426 (5)	
C6	1.1309 (4)	0.4206 (4)	0.22997 (8)	0.0515 (7)	
H6A	1.0054	0.3804	0.2365	0.062*	
H6B	1.2134	0.3195	0.2281	0.062*	
C7	1.1199 (4)	0.5162 (4)	0.19417 (8)	0.0453 (6)	
H7A	1.0920	0.4343	0.1747	0.054*	
H7B	1.0190	0.6026	0.1945	0.054*	
C8	1.2147 (3)	0.7874 (3)	0.12720 (7)	0.0367 (5)	
C9	1.2684 (4)	0.9644 (3)	0.13041 (8)	0.0443 (6)	
H9A	1.3834	1.0013	0.1407	0.053*	
C10	1.1341 (4)	1.0751 (3)	0.11683 (8)	0.0486 (6)	
H10A	1.1467	1.1961	0.1166	0.058*	
C11	1.0374 (3)	0.7667 (3)	0.11097 (6)	0.0366 (5)	
C12	0.9260 (3)	0.6133 (3)	0.10159 (6)	0.0359 (5)	
C13	0.7017 (3)	0.4705 (3)	0.07619 (7)	0.0367 (5)	
C14	0.5288 (3)	0.4345 (3)	0.05505 (6)	0.0374 (5)	
C15	0.4063 (4)	0.5660 (4)	0.04346 (8)	0.0492 (6)	
H15A	0.4393	0.6814	0.0484	0.059*	
C16	0.1984 (4)	0.3691 (4)	0.01865 (8)	0.0541 (7)	
H16A	0.0842	0.3453	0.0064	0.065*	
C17	0.3143 (3)	0.2309 (4)	0.02888 (7)	0.0442 (6)	
C18	0.4814 (3)	0.2626 (3)	0.04777 (7)	0.0415 (5)	
H18A	0.5600	0.1713	0.0554	0.050*	

### Atomic displacement parameters (Å^2^)

**Table:**

	*U*^11^	*U*^22^	*U*^33^	*U*^12^	*U*^13^	*U*^23^
S1	0.0598 (4)	0.0272 (3)	0.0493 (4)	0.0054 (2)	−0.0094 (3)	0.0027 (3)
S2	0.0358 (3)	0.0367 (3)	0.0390 (3)	0.0038 (2)	0.0026 (2)	−0.0008 (2)
S3	0.0600 (4)	0.0788 (5)	0.0392 (4)	0.0036 (4)	0.0025 (3)	0.0054 (4)
Br1	0.04669 (17)	0.0620 (2)	0.0854 (3)	−0.00962 (13)	−0.01522 (15)	−0.01519 (17)
O1	0.0451 (9)	0.0303 (7)	0.0356 (9)	0.0030 (6)	−0.0073 (7)	0.0014 (7)
O2	0.0351 (9)	0.0595 (12)	0.0571 (12)	−0.0017 (8)	0.0089 (8)	0.0006 (9)
O3	0.0534 (11)	0.0408 (9)	0.0449 (11)	0.0115 (8)	0.0011 (8)	−0.0084 (8)
N1	0.0554 (13)	0.0304 (10)	0.0638 (15)	−0.0004 (9)	−0.0274 (11)	0.0006 (10)
N2	0.0576 (13)	0.0280 (9)	0.0671 (16)	−0.0017 (9)	−0.0277 (12)	−0.0001 (10)
N3	0.0317 (9)	0.0376 (10)	0.0379 (11)	−0.0042 (7)	−0.0015 (8)	−0.0015 (8)
N4	0.0522 (14)	0.0607 (15)	0.0610 (16)	0.0138 (11)	−0.0163 (12)	0.0063 (12)
C1	0.0475 (14)	0.0396 (12)	0.0459 (15)	−0.0084 (10)	0.0007 (11)	−0.0064 (11)
C2	0.0303 (11)	0.0482 (13)	0.0404 (14)	0.0004 (9)	−0.0031 (10)	−0.0065 (11)
C3	0.0443 (14)	0.0685 (19)	0.0534 (18)	−0.0038 (13)	−0.0043 (13)	−0.0197 (15)
C4	0.0549 (16)	0.087 (2)	0.0439 (17)	0.0084 (15)	−0.0062 (13)	−0.0193 (16)
C5	0.0370 (12)	0.0512 (14)	0.0395 (14)	0.0016 (10)	−0.0015 (10)	0.0004 (11)
C6	0.0537 (15)	0.0537 (15)	0.0471 (16)	−0.0187 (12)	0.0028 (12)	0.0018 (13)
C7	0.0367 (12)	0.0570 (16)	0.0420 (14)	−0.0119 (11)	−0.0036 (10)	−0.0012 (12)
C8	0.0431 (12)	0.0300 (10)	0.0369 (13)	0.0003 (9)	0.0010 (10)	0.0013 (9)
C9	0.0537 (15)	0.0346 (11)	0.0445 (15)	−0.0070 (10)	−0.0015 (12)	−0.0003 (11)
C10	0.0658 (17)	0.0285 (11)	0.0512 (16)	−0.0034 (11)	−0.0048 (13)	0.0019 (11)
C11	0.0484 (13)	0.0253 (9)	0.0358 (13)	0.0025 (9)	−0.0029 (10)	0.0002 (9)
C12	0.0454 (12)	0.0281 (10)	0.0338 (12)	0.0029 (9)	−0.0075 (10)	−0.0024 (9)
C13	0.0427 (12)	0.0313 (10)	0.0357 (12)	0.0030 (9)	−0.0055 (10)	−0.0023 (9)
C14	0.0410 (12)	0.0401 (12)	0.0308 (12)	0.0033 (10)	−0.0042 (9)	0.0000 (10)
C15	0.0543 (15)	0.0456 (14)	0.0475 (16)	0.0079 (12)	−0.0071 (12)	0.0021 (12)
C16	0.0401 (13)	0.0674 (19)	0.0542 (17)	0.0042 (13)	−0.0107 (12)	0.0033 (15)
C17	0.0385 (12)	0.0524 (14)	0.0415 (14)	0.0002 (11)	−0.0056 (11)	−0.0016 (12)
C18	0.0373 (12)	0.0436 (12)	0.0432 (14)	0.0049 (10)	−0.0076 (10)	−0.0003 (11)

### Geometric parameters (Å, °)

**Table:**

S1—C10	1.698 (3)	C3—C4	1.345 (4)
S1—C11	1.720 (2)	C3—H3A	0.9300
S2—O3	1.4233 (19)	C4—H4A	0.9300
S2—O2	1.4248 (18)	C5—C6	1.496 (4)
S2—N3	1.625 (2)	C6—C7	1.512 (4)
S2—C8	1.774 (2)	C6—H6A	0.9700
S3—C4	1.707 (4)	C6—H6B	0.9700
S3—C5	1.718 (3)	C7—H7A	0.9700
Br1—C17	1.884 (3)	C7—H7B	0.9700
O1—C13	1.353 (3)	C8—C11	1.381 (3)
O1—C12	1.362 (3)	C8—C9	1.410 (3)
N1—C13	1.284 (3)	C9—C10	1.356 (4)
N1—N2	1.394 (3)	C9—H9A	0.9300
N2—C12	1.291 (3)	C10—H10A	0.9300
N3—C1	1.473 (3)	C11—C12	1.448 (3)
N3—C7	1.479 (3)	C13—C14	1.456 (3)
N4—C15	1.329 (4)	C14—C18	1.381 (3)
N4—C16	1.329 (4)	C14—C15	1.386 (4)
C1—C2	1.501 (4)	C15—H15A	0.9300
C1—H1A	0.9700	C16—C17	1.382 (4)
C1—H1B	0.9700	C16—H16A	0.9300
C2—C5	1.349 (4)	C17—C18	1.375 (3)
C2—C3	1.417 (4)	C18—H18A	0.9300
			
C10—S1—C11	92.19 (12)	N3—C7—C6	108.8 (2)
O3—S2—O2	119.45 (11)	N3—C7—H7A	109.9
O3—S2—N3	107.45 (11)	C6—C7—H7A	109.9
O2—S2—N3	106.69 (11)	N3—C7—H7B	109.9
O3—S2—C8	110.45 (11)	C6—C7—H7B	109.9
O2—S2—C8	105.95 (11)	H7A—C7—H7B	108.3
N3—S2—C8	106.06 (11)	C11—C8—C9	112.6 (2)
C4—S3—C5	91.51 (15)	C11—C8—S2	129.09 (17)
C13—O1—C12	102.63 (17)	C9—C8—S2	118.17 (19)
C13—N1—N2	106.4 (2)	C10—C9—C8	112.7 (2)
C12—N2—N1	106.31 (19)	C10—C9—H9A	123.7
C1—N3—C7	114.6 (2)	C8—C9—H9A	123.7
C1—N3—S2	117.19 (16)	C9—C10—S1	112.13 (19)
C7—N3—S2	117.26 (16)	C9—C10—H10A	123.9
C15—N4—C16	117.9 (2)	S1—C10—H10A	123.9
N3—C1—C2	109.1 (2)	C8—C11—C12	132.5 (2)
N3—C1—H1A	109.9	C8—C11—S1	110.41 (17)
C2—C1—H1A	109.9	C12—C11—S1	117.10 (17)
N3—C1—H1B	109.9	N2—C12—O1	112.0 (2)
C2—C1—H1B	109.9	N2—C12—C11	130.1 (2)
H1A—C1—H1B	108.3	O1—C12—C11	117.87 (19)
C5—C2—C3	112.2 (3)	N1—C13—O1	112.6 (2)
C5—C2—C1	122.4 (2)	N1—C13—C14	126.3 (2)
C3—C2—C1	125.3 (2)	O1—C13—C14	121.0 (2)
C4—C3—C2	113.0 (3)	C18—C14—C15	119.0 (2)
C4—C3—H3A	123.5	C18—C14—C13	118.6 (2)
C2—C3—H3A	123.5	C15—C14—C13	122.3 (2)
C3—C4—S3	111.7 (2)	N4—C15—C14	123.0 (3)
C3—C4—H4A	124.2	N4—C15—H15A	118.5
S3—C4—H4A	124.2	C14—C15—H15A	118.5
C2—C5—C6	124.5 (2)	N4—C16—C17	122.6 (3)
C2—C5—S3	111.6 (2)	N4—C16—H16A	118.7
C6—C5—S3	124.0 (2)	C17—C16—H16A	118.7
C5—C6—C7	108.6 (2)	C18—C17—C16	119.7 (3)
C5—C6—H6A	110.0	C18—C17—Br1	119.8 (2)
C7—C6—H6A	110.0	C16—C17—Br1	120.4 (2)
C5—C6—H6B	110.0	C17—C18—C14	117.8 (2)
C7—C6—H6B	110.0	C17—C18—H18A	121.1
H6A—C6—H6B	108.3	C14—C18—H18A	121.1
			
C13—N1—N2—C12	−0.5 (3)	C8—C9—C10—S1	0.5 (3)
O3—S2—N3—C1	170.10 (17)	C11—S1—C10—C9	−0.6 (2)
O2—S2—N3—C1	40.9 (2)	C9—C8—C11—C12	179.0 (3)
C8—S2—N3—C1	−71.75 (19)	S2—C8—C11—C12	−5.7 (4)
O3—S2—N3—C7	−47.6 (2)	C9—C8—C11—S1	−0.3 (3)
O2—S2—N3—C7	−176.79 (18)	S2—C8—C11—S1	174.96 (15)
C8—S2—N3—C7	70.6 (2)	C10—S1—C11—C8	0.5 (2)
C7—N3—C1—C2	45.9 (3)	C10—S1—C11—C12	−178.9 (2)
S2—N3—C1—C2	−170.77 (16)	N1—N2—C12—O1	0.1 (3)
N3—C1—C2—C5	−13.3 (3)	N1—N2—C12—C11	179.3 (3)
N3—C1—C2—C3	170.1 (2)	C13—O1—C12—N2	0.3 (3)
C5—C2—C3—C4	0.0 (4)	C13—O1—C12—C11	−179.0 (2)
C1—C2—C3—C4	176.9 (3)	C8—C11—C12—N2	15.1 (5)
C2—C3—C4—S3	0.0 (3)	S1—C11—C12—N2	−165.6 (2)
C5—S3—C4—C3	0.0 (2)	C8—C11—C12—O1	−165.8 (2)
C3—C2—C5—C6	179.2 (3)	S1—C11—C12—O1	13.5 (3)
C1—C2—C5—C6	2.2 (4)	N2—N1—C13—O1	0.7 (3)
C3—C2—C5—S3	0.0 (3)	N2—N1—C13—C14	−177.0 (2)
C1—C2—C5—S3	−177.00 (19)	C12—O1—C13—N1	−0.6 (3)
C4—S3—C5—C2	0.0 (2)	C12—O1—C13—C14	177.3 (2)
C4—S3—C5—C6	−179.2 (2)	N1—C13—C14—C18	−14.6 (4)
C2—C5—C6—C7	−20.7 (4)	O1—C13—C14—C18	167.9 (2)
S3—C5—C6—C7	158.4 (2)	N1—C13—C14—C15	162.4 (3)
C1—N3—C7—C6	−67.1 (3)	O1—C13—C14—C15	−15.1 (4)
S2—N3—C7—C6	149.6 (2)	C16—N4—C15—C14	−0.2 (4)
C5—C6—C7—N3	49.6 (3)	C18—C14—C15—N4	0.2 (4)
O3—S2—C8—C11	29.5 (3)	C13—C14—C15—N4	−176.8 (3)
O2—S2—C8—C11	160.2 (2)	C15—N4—C16—C17	−0.7 (5)
N3—S2—C8—C11	−86.6 (2)	N4—C16—C17—C18	1.6 (4)
O3—S2—C8—C9	−155.4 (2)	N4—C16—C17—Br1	−178.6 (2)
O2—S2—C8—C9	−24.7 (2)	C16—C17—C18—C14	−1.5 (4)
N3—S2—C8—C9	88.4 (2)	Br1—C17—C18—C14	178.66 (19)
C11—C8—C9—C10	−0.1 (3)	C15—C14—C18—C17	0.7 (4)
S2—C8—C9—C10	−175.9 (2)	C13—C14—C18—C17	177.8 (2)

### Hydrogen-bond geometry (Å, °)

**Table:**

*D*—H···*A*	*D*—H	H···*A*	*D*···*A*	*D*—H···*A*
C7—H7A···N2	0.97	2.52	3.283 (4)	136
C10—H10A···O3^i^	0.93	2.49	3.330 (3)	150
C10—H10A···N2^i^	0.93	2.42	3.183 (3)	139

Symmetry codes: (i) *x*, *y*+1, *z*.
